# Editorial: Mechanistic and physiological implications of insulin resistance in metabolic diseases

**DOI:** 10.3389/fendo.2024.1446492

**Published:** 2024-07-03

**Authors:** Hilda E. Ghadieh, Marco Infante, Carlos H. Sponton

**Affiliations:** ^1^ Department of Biomedical Sciences, Faculty of Medicine and Medical Sciences, University of Balamand, Tripoli, Lebanon; ^2^ Section of Diabetes & Metabolic Disorders, UniCamillus, Saint Camillus International University of Health Sciences, Rome, Italy; ^3^ Department of Structural and Functional Biology, Institute of Biology, University of Campinas, São Paulo, Brazil; ^4^ Obesity and Comorbidities Research Center (OCRC), Institute of Biology, University of Campinas, São Paulo, Brazil

**Keywords:** metabolic diseases, metabolic syndrome, insulin signaling, insulin resistance, diabetes mellitus, obesity, endocrinology, metabolism

The present Research Topic entitled “*Mechanistic and Physiological Implications of Insulin Resistance in Metabolic Diseases*” aimed to collect the most updated reports in the field of insulin resistance (IR) and metabolic syndrome (MetS). Since the pathophysiological mechanisms of IR remain only partly understood, we herein present a comprehensive understanding of insulin signaling regulation by providing new insights into the impact of altered insulin action on metabolic processes.

A cross-sectional study (Wu et al.) investigated the relationship between triglyceride glucose-body mass index (TyG-BMI) and testosterone levels in adult males, considering TyG-BMI as a novel marker of IR. Using data from the NHANES 2011–2016, the analysis revealed a negative association between TyG-BMI and circulating testosterone levels, even after adjusting for confounders. Testosterone levels were significantly lower in the groups with higher TyG-BMI values. Thus, the ability of the TyG-BMI index to predict testosterone deficiency surpassed that of both the homeostatic model assessment for insulin resistance (HOMA-IR) index and the triglyceride-glucose (TyG) index.

Another cross-sectional study (Kim et al.) explored a practical approach for diagnosing metabolic dysfunction-associated fatty liver disease (MAFLD). While the exact pathophysiology of MAFLD remains uncertain, IR is a main contributor. Analyzing databases of regular health check-up examinations, researchers investigated the association between MAFLD and four indices, namely: TyG index, fatty liver index (FLI), and modified TyG-related parameters such as TyG-BMI and TyG-waist circumference (TyG-WC). Authors found that modified TyG-related parameters were strongly associated with MAFLD, showing an even greater predictive power as compared to the TyG index. Therefore, modified TyG indices may offer reliable tools for MAFLD prediction in daily clinical practice, simplifying diagnosis and improving patient care in real-world settings.

A meta-analysis (Liu et al.) aimed to evaluate the impact of mind-body exercise interventions on patients with MetS, focusing on factors such as IR. Fourteen randomized controlled trials (RCTs) involving 1148 patients were analyzed. Results showed that mind-body exercise significantly improved various metabolic parameters, including IR, WC, BMI, blood pressure, fasting blood glucose, triglycerides (TG), and high-density lipoprotein (HDL) cholesterol. Subgroup analysis revealed that interventions with fitness qigong, lasting 24–48 weeks and performed 6–7 times per week, were particularly effective in improving risk factors among patients with MetS. The study concluded that mind-body exercise is beneficial for patients with MetS, recommending low-to moderate-intensity fitness qigong interventions.

A retrospective cross-sectional study (Ye et al.) explored the relationship between IR, hyperinsulinemia, and bone mineral density (BMD) in 437 non-diabetic postmenopausal women. Results showed that elevated HOMA-IR and fasting insulin levels were associated with increased BMD and decreased follicle-stimulating hormone (FSH) values in non-diabetic postmenopausal women. These findings suggest a potential mediating role of IR in FSH-induced BMD suppression in non-diabetic postmenopausal women, leading to a deeper understanding of the mechanisms underlying the BMD decline in postmenopausal women. Thus, it is important to explore strategies for regulating glucose metabolism within an optimal range to promote metabolic and bone health in this population.

Another study (Alanazi et al.) investigated the correlation between KLF14 rs4731702 single nucleotide polymorphism (SNP) and the risk of type 2 diabetes mellitus (T2DM) and dyslipidemia across various ethnic groups. Three study groups - healthy subjects, patients with T2DM, and patients with cardiometabolic disorders - underwent biochemical analysis for glycemic and lipid biomarkers, along with genotyping for KLF14 rs4731702 SNP using the Tetra ARMS-PCR method. Results revealed that KLF14 rs4731702 is associated with altered glycemic biomarkers and lipid profile in T2DM patients. Specifically, individuals with the C allele exhibited higher IR and a worse lipid profile as compared to the T allele carriers. This study also found that the prevalence of KLF14 rs4731702 SNP is higher in women and increases with age, emphasizing its role in the pathogenesis of T2DM and cardiovascular disease.

Pulmonary arterial hypertension (PAH) is a severe vascular disease characterized by elevated pulmonary blood pressure and subsequent development of heart failure. Despite advances in therapeutic options, PAH remains associated with a substantially high mortality. The complex PAH pathogenesis involves various pathways leading to vascular inflammation and anatomical changes that are not limited to the lungs. Recent studies have highlighted disturbances in glucose metabolism in PAH patients, suggesting a potential link of PAH with IR. The current review (Zanotto et al.) explored the potential molecular links between PAH and IR, including a reduction in circulating adiponectin and a decrease in perivascular adiponectin expression in the pulmonary artery, which have been associated with subclinical inflammation causing reduced expression of peroxisome proliferator-activated receptor gamma (PPAR-γ) and bone morphogenetic protein receptor type 2 (BMPR2). Consequently, such molecular mechanisms induce mitochondrial dysfunction and endoplasmic reticulum (ER) stress. Thus, targeting these molecular pathways may pave the way for the development of novel therapeutic options for the treatment of PAH.

The discovery of insulin in 1921 marked a new era for research on insulin activity and insulin resistance, leading to advancements in the diagnosis and treatment of IR-related diseases. A mini review (Le et al.) synthesized recent scientific discoveries to better elucidate the mechanisms of insulin action and enable innovative applications in the field of IR. This article also examined treatment strategies for obesity, cardiovascular disease, Alzheimer’s disease and cancer, based on a better understanding of the mechanisms underlying IR in these conditions.

An opinion article (Zamolodchikova et al.) provided a new perspective on the multifaceted effects of insulin on metabolism and cell function, emphasizing its role in glucose uptake, glycogen synthesis and cell growth. Disruption of the insulin signaling pathways leads to IR, which is highly prevalent in conditions such as T2DM and obesity. The renin-angiotensin system (RAS) also influences insulin sensitivity, with angiotensin II (ANGII) altering insulin signaling. Insulin directly regulates RAS function, and impaired insulin signaling may lead to RAS dysfunction, thus contributing to diabetes complications. A better comprehension of the interplay between RAS and insulin may offer new insights into the pathophysiology of IR and its related cardiometabolic comorbidities.

We thank all the Authors who contributed to the present Research Topic, which undoubtedly provided novel insights into the pathophysiological role of IR in various chronic non-communicable diseases and may prompt researchers to investigate the potential therapeutic role of insulin-sensitizing agents in these conditions.

## Author contributions

HEG: Conceptualization, Writing – original draft, Writing – review & editing. MI: Writing – review & editing. CHS: Writing – review & editing.

